# Aspects of the Complement System in New Era of Xenotransplantation

**DOI:** 10.3389/fimmu.2022.860165

**Published:** 2022-04-14

**Authors:** Shuji Miyagawa, Akira Maeda, Chiyoshi Toyama, Shuhei Kogata, Chizu Okamatsu, Riho Yamamoto, Kazunori Masahata, Masafumi Kamiyama, Hiroshi Eguchi, Masahito Watanabe, Hiroshi Nagashima, Masahito Ikawa, Katsuyoshi Matsunami, Hiroomi Okuyama

**Affiliations:** ^1^ Department of Pediatric Surgery, Osaka University Graduate School of Medicine, Osaka, Japan; ^2^ International Institute for Bio-Resource Research, Meiji University, Kanagawa, Japan; ^3^ Research Institute for Microbial Diseases, Osaka University, Osaka, Japan; ^4^ Department of Pharmacognosy, Graduate School of Biomedical and Health Sciences, Hiroshima University, Hiroshima, Japan

**Keywords:** complement-immunological terms, complement regulatory protein, complement receptor, gene-modified pigs, locally produced complement, non-Gal antigens

## Abstract

After producing triple (Gal, H-D and Sd^a^)-KO pigs, hyperacute rejection appeared to no longer be a problem. However, the origin of xeno-rejection continues to be a controversial topic, including small amounts of antibodies and subsequent activation of the graft endothelium, the complement recognition system and the coagulation systems. The complement is activated via the classical pathway by non-Gal/H-D/Sda antigens and by ischemia-reperfusion injury (IRI), via the alternative pathway, especially on islets, and via the lectin pathway. The complement system therefore is still an important recognition and effector mechanism in xeno-rejection. All complement regulatory proteins (CRPs) regulate complement activation in different manners. Therefore, to effectively protect xenografts against xeno-rejection, it would appear reasonable to employ not only one but several CRPs including anti-complement drugs. The further assessment of antigens continues to be an important issue in the area of clinical xenotransplantation. The above conclusions suggest that the expression of sufficient levels of human CRPs on Triple-KO grafts is necessary. Moreover, multilateral inhibition on local complement activation in the graft, together with the control of signals between macrophages and lymphocytes is required.

## Introduction

Last year, kidneys from αGal (1) -knock out (KO) pigs were transplanted into a brain-dead patient, and the results revealed that hyper-acute rejection could be overcome by genetic modification. The heart from a pig in which genes-modified was transplanted into a patient with end-stage heart failure. These events show that clinical application of xenografts has finally begun. Since there are many review articles on complement and xenotransplantation (2–4), we leave the general discussion to them and focus mainly on the recent progress made in this area.

The first breakthrough in xenotransplantation research was the report of species differences in the complement system (5). This was combined with transgenic technology and led to the creation of the complement regulatory proteins (CRPs)-transgenic pig (6, 7). The next breakthrough was the discovery of heterologous carbohydrate antigens. Knockout technology was added to nuclear transfer technology to produce KO pigs. Further studies with the goal of elucidating the relationship between complement and the coagulation system, reperfusion injury, etc in transplantation. In addition, a new topic that is generally associated with transplantation has arisen, namely, the local production of complement in the graft and complement receptors (8). These issues will become more apparent and may also be relevant to Xenotransplantation.

## Features of Complement Regulatory Proteins

**Figure 1:**
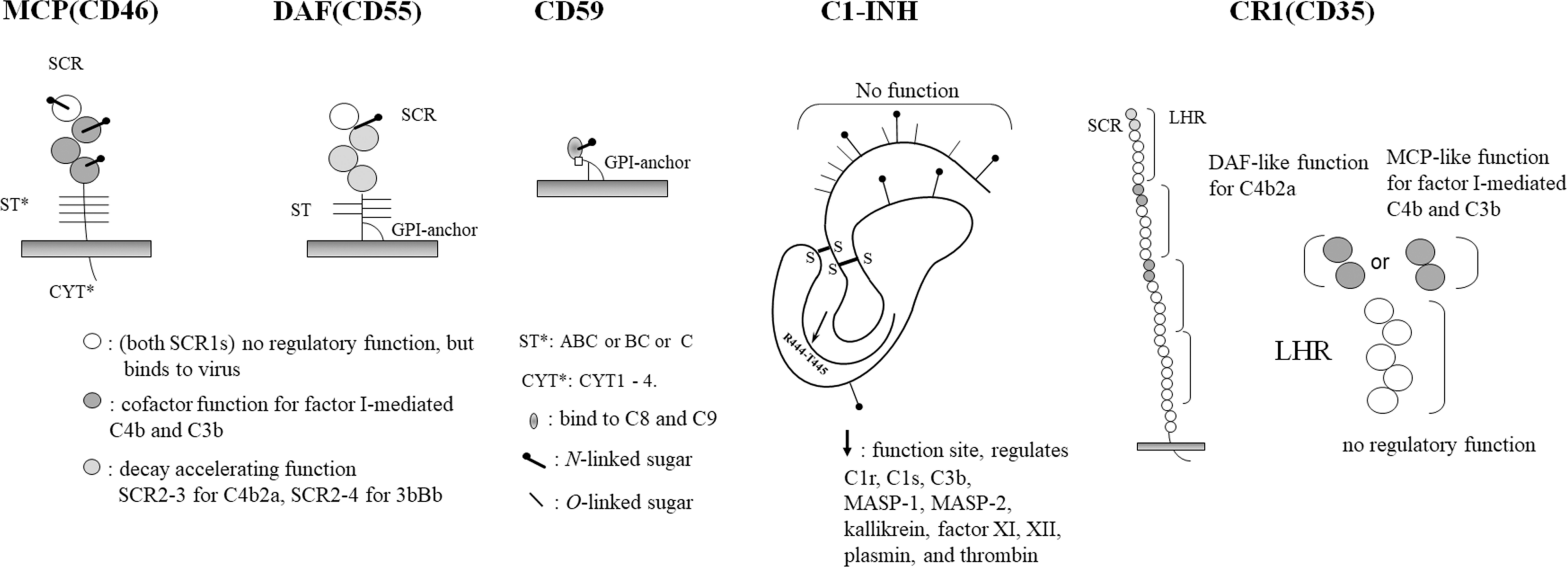
Complement regulatory proteins. A schematic diagram of each complement regulatory proteins, MCP, DAF, CD59, C1-INH and CR1, are shown.

Regarding complement regulatory proteins, CD46, CD55, and CD59 are currently the focus of attention, and it is recommended that at least two of these be expressed in grafts. Since these functions are all different, expressing all of them would be the best strategy. In addition, C1 esterase inhibitor (C1-INH) has been used as a drug by some groups, but due to its wide range of functions, it would be better to be expressed in grafts using the membrane form (9) (**Figure 1**).

### Membrane Cofactor Protein; CD46

MCP is a single-chain glycoprotein with four short onsensus repeats (SCRs), an Ser/Thr-rich (ST) region (ABC, BC and C) and four types of cytoplasmic tails (CYT1 to CYT4). As a function, it is a cofactor for factor I in serum, an irreversible reaction that restricts the degradation of C3b to iC3b and C4b to C4d, which are components of C3 convertase that is formed on the same membrane. The sites involved in the binding and degradation of both enzymes are SCR2 to SCR4, and the N-glycans of SCR2 and SCR4 are required for their function (10, 11).

In addition to the complementary functions of the cell surface, the expression of MCP is intimately involved in the regulation of T cell activation.

During T cell activation, CD46 is mobilized in the immune synapse, and its ST-region is required. The mobilization of CD46 to immune synapses causes T cells to switch from producing the proinflammatory cytokine interferon-γ (IFN-γ) (Th1) to producing IL-10 (Tr1), and these reactions are related to CYT1 and CYT2 (12).

### Decay-Accelerating Factor; CD55 

DAF consists of four SCRs and ST regions, and a glycosyl phosphatidyl inositol (GPI)-anchor and is expressed on most blood cells. SS-bound dimers are present in the DAF of blood cells.

In addition, there is also a water-soluble form of DAF as a splicing variant.

DAF promotes the dissociation of the C3 convertases C4b2a and C3Bb, SCR2 and SCR3 are responsible for the dissociation of C4b2a, and SCR2-SCR4 are responsible for the dissociation of C3Bb (13–15).

Moreover, in addition to the complement system, DAF binds to CD97 on T cells via SCR1, thus controlling their activation and the differentiation of Type 1 regulatory T cells (Tr1 cells).

It also directly inhibits the cytotoxic function of Natural Killer (NK) cells by SCR2-4 (16).

### CD59 (Membrane-Attack Complex Inhibition Factor, Homologous Restriction Factor or Protection)

CD59 is a relatively small glycoprotein that contains a GPI-anchor (17). It is a member of the neurotoxin family and is also classified as a member of the Ly6 superfamily (18). As a function, it binds to C8 and C9 to inhibit the formation of membrane attack complexes (MACs). N-glycans are not required for its function, but, rather, act in an inhibitory manner.

This molecule is also expressed on T cells and is associated with CD58, which is involved in the adhesion and activation of T cells. CD59 on T cells is reported to function as a signal transducing molecule for activation (19).

### C1 Esterase Inhibitor

This molecule inhibits the action of serine proteases C1r (activates C1s) and C1s (activate C4 and C2). The active center is located in the Val-Ala-Arg-Thr-Leu portion of the C-terminal side, which is truncated at Arg(444) and binds to the active serine of C1r and C1s, where it directly and irreversibly inhibits their enzymatic activities. The peptide portion from the N-terminus to 101 is not involved in this function (20–22).

Importantly, this molecule also inhibits the activities of other serine proteases such as kallikrein, plasmin, and the coagulation factors XI and XII. For the above reasons, this molecule is sometimes used as a drug for xenotransplantation (9).

### Complement Receptor Type 1

There are four allotypes (A-D) of CR1. The basic primary structure is that of a type 1 transmembrane glycoprotein with four long homologous repeats (LHRs) consisting of seven SCRs, which are linked to the transmembrane portion by two other SCRs. It combines the dissociation activity of the C3-converting enzymes C4b2a and C3bBb, and the degradation activity of C4b to C4d and C3b to C3dg.

As a feature, this molecule functions extrinsically, so it is not suitable for being transgenic in the original molecule.

CR1 on monocytes and macrophages also functions as a receptor that binds to C3b molecules on the surface of foreign substances to induce phagocytosis. CR1 on erythrocytes binds to C3b-bound (opsonized) bacteria, viruses, and immune complexes and transports them to the monocyte-macrophage system. CR1 on B-lymphocytes cooperates with complement receptor type 2 (CR2) to activate lymphocytes, while CR2 prevents these phagocytes from overreactin (23–26).

### Thrombomodulin 

This molecule is basically an anticoagulant factor, and promotes the activation of protein C with thrombin. In addition, thrombomodulin binds to factor H, thereby promoting the inactivation of complement C3b. or inactivating C3a and C5a via an activated thrombin activatable fibrinolysis inhibitor (TAFI) (27–29).

### A Bound Form of Serum CRP

Serum contains several SCRs, including C4bp (30) and FactorH (31). These molecules show DAF-like catalytic functions as well as MCP-like cofactor functions. Therefore, there are reports of converting these serum factors into membrane forms and studying their functions on the cell surface.

As a result, these molecules show MCP and/or DAF functions on porcine cells. However, concerning membrane-type factor I, it functions in vitro, but its *in vivo* function when converted to membrane form is currently unknown.

These are synthetic molecules based on SCR2-4 hybrids of MCP and DAF, and are considered to be suitable for use in transgenic pigs. In addition, C1 Estarase Inhibitor *Thrombomodulin+DAF+MCP (CTDM or CDM) hybrids were reported (2, 32, 33).

### Functions Other than Complement Control—Virus Receptors

It has been reported that CR functions as a receptor for a number of viruses. One of the solutions for preventing viral adhesion is to remove the SCR1 of MCP & DAF. This may not completely eliminate the function as virus receptor, but it may greatly reduce this function (34–36).

## Transgenic Pigs

The trend appears to be that at least two of the CRPs, MCP, DAF (6), and CD59, are transgenic in pigs. This is reasonable since the function of each molecule is different from the other (**Table 1**) (2, 37–51).

**Table 1:** Report of CRP transgenic pig heart and kidney transplantation into primates, and comparison with Gal-KO. In addition, the best record for reference.

I. Heart (Hetero)				
Pigs	Recipient	Survival days	Immunosuppression	Report
CD46	Baboon	1h – 16 (5.25)	none	Adams et al. (38)
CD46	Baboon	15 – 137 (96)	Rituximab, ATG, TPC, FK506, Rapa, CS	McGregor et al. (39)
CD55	Baboon	(5.1)	none	Thompson (40)
CD55	Baboon	4 – 139 (27)	GAS914, ATG, sCR1, LoCD2b, Thymic irradiation, MMF, CVF, anti-CD154, CS	Houser et al. (41)
CD59	Baboon	2.25 – 3.0 h.	none	Diamond et al. (42)
Gal-KO	Baboon	16 – 179 (78)	ATG, LoCD2b, Thymic irradiation, CVF, anti-CD154, MMF, CS	Kuwaki et al. (43)
Gal-KO with CD46 and TBM	Baboon	Max 2.5 years	Anti-CD20, ATG, anti-CD40, CVF, CS	Muhiuddin et al. (44)
**II. Heart (Ortho)**				
**Pigs**	**Recipient**	**Survival days**	**Immunosuppression**	**Report**
CD46	Baboon	34 - 57 (40)	αGal-polymer, anti-CD20, ATG, FK506, Rapa, CS	McGregor et al. (45)
CD55	Baboon	39	CyP, CsA, MMF, CS	Vial (46)
Gal/β4GalNT2/GHR-KO with CD46, TBM, EPCR and CD47	Baboon	182, 264	Anti-CD20, ATG, anti-CD40, CVF, CS	Muhiuddin*
**III. Kidney**				
**Pigs**	**Recipient**	**Survival days**	**Immunosuppression**	**Report**
CD46	Baboon	13 – 15 (14)	CsA, CyP, CS, splenectomy, affinity colum for anti-Gal Ab	Dean et al. (47)
CD55	Cynomolgus	5 – 78 (35)	CsA, CyP, CS, splenectomy	Cozzi et al. (48)
CD55	Baboon	21 – 36 (23)	GAS914, CyP, ATG, CsA, Rapa, CS	Ghanekar et al. (49)
Gal-KO	Baboon	20 – 34 (29)	ATG, LoCD2b, CVF, anti-CD154, MMF, CS	Yamada et al. (50)
Gal-KO with CD55	Rhesus	>70 – 499 (328)	anti-CD4mAb, anti-Cd154mAb, MMF, CS	Kim et al. (51)

ATG, anti-thymocyte globulin; TPC, anti-Gal-polyethyleneglycol conjugate; Rapa, rapamycin; CS, steroid; GAS914, a soluble Gal (α1-3) Gal polymer; CVF, cobra venom factor; LoCD2b, rat anti-primate CD2b monoclonal antibody; sCR1, soluble complement receptor type I;

MMF, mycophenolate mofetil; CsA, cyclosporine; CyP, cyclophosphamide.

* : Mohiuddin MM, 215.4 Select Life-Supporting Multi-Gene Cardiac Xenografts from Swine Demonstrate Survival >8 months in Baboons, with implications for human Clinical Trials. The Joint Congress of the International Xenotransplantation Association (IXA) and the Cell Transplant and Regenerative Medicine Society (CTRMS), taking place virtually. September 23-25, 2021.

To elaborate, DAF is predominant in its catalytic function in vitro, but leaves remnants of C4b and C3b on the cell surface. This does not affect the opsonin function of immune cells, such as macrophages, which produce complement receptors. On the other hand, MCP may be inferior to DAF in the speed at which it controls complement from the cell membrane, but the cofactor action of Factor I allows it to completely remove complement from the membrane. Regarding CD59, as described in the coagulation factors section, some of the complement is activated from C5 on the porcine cell membrane, so it is necessary to express CD59 in order to inhibit MAC formation. However, since the CD59 molecule is small and does not have much species-specific heterogeneity, pig CD59 is also considered to be functional. Therefore, using human CD59 is not absolutely necessary (52).

On the other hand, the question arises as to how hMCP is expressed in pigs. MCP contains a part of a DNA molecule that negatively regulates its expression, which initially caused some problems. The ST region of MCP is related to its function, and the CYT part is related to the expression of metabolism, i.e., the amount of expression (53, 54).

In addition, if human MCPs are expressed in pig T cells, this might be a problem, but no information regarding this is available. However, the development of a δ-CYT type MCP-pig has been considered.

## Complement Receptors

### CR2(CD21)

This molecule is mainly produced on B cells, binds to C3dg and C3dg and regulates the function of B cells.

Normally, antigens alone have a weak ability to be presented to B cells. However, when complement is attached to the antigen, the CR2 that is expressed on B cells recognizes the degradation products derived from complement, which induces a second signal-like reaction in T cells and helps them to recognize the antigen. In addition, another function has been identified for the CR2-mediated role of follicular DCs in the germ center of lymph nodes for B cells (55).

### CR3 (CD11b/CD18, Mac1)

This is localized on granulocytes, macrophages, NK cells, related to phagocytosis.

### CR4 (CD11c/CD18, p150,95)

This is localized on granulocytes, macrophages, dendritic cells, and is related to monocyte migration.

### C3a/C4a Receptors

### C5a Receptors (CD88)

### C1q Receptors (gC1q, cC1q)

These molecules are related to the increased phagocytosis of monocytes/macrophages, the increased oxidative metabolism of neutrophils, and the regulation of antibody production in B cells (56).

### CRIg

CRIg binds to C3b, iC3b, and C3c. In T cells, it is involved in their proliferation and the release of cytokines, is expressed on DCs and is involved in immunosuppression (57).

## Role of Locally Produced Complement and Complosome

### Locally Synthesized Complement

In the allo transplantation, locally synthesized complement components have a profound effect on the grafts. A well-known example of this is a mouse kidney transplant model, where recipients that received C3-/-kidneys showed long-term graft survival (8). Moreover, a C5aR deficiency and inhibition has also been reported to prolong the survival of renal and intestinal allogeneic grafts, reduce apoptosis and attenuate the infiltration of inflammatory cells (58, 59). As research has progressed, it has become clear that the relationship between C3a and C3 receptors (C3aR) and C5a and C5 receptors (C5aR) plays a major role in the presentation of antigen-presenting cells (APCs) to T cells, and that these responses and stimuli enter T cells as a strong auxiliary signal. On the other hand, the expression of complement regulators on the surface of the graft has also been shown to be important and is possibly related to graft survival (60).

It is conceivable that a similar reaction occurs between graft-infiltrated APC-T cells with the complement they produce and their receptors in the xenograft.

It might be necessary to verify whether the porcine complement produced by xenografts reacts with human complement receptors, and the possibility that the local porcine complement could attack human immune cells, in addition to the case of liver transplantation (61).

### Complosome

It is generally accepted that complement activation occurs intracellularly (60). Specifically, human CD4 + T cells express C3aR not only on the cell surface but also on lysosomes. It has also been reported that C5 is conserved in human T cells. When T cells are activated, intracellular C5a binds to intracellular C5aR1, resulting in the activation of endogenous NOD-, LRR- and pyrin domain-containing protein 3 (NLRP3) inflammasomes, and the secretion of the autocrine IL-1β. CD4+ T cells express C5aR2 on their surface and intracellularly, and negatively regulate C5aR1-driven NLRP3 inflammasome activity (62). Unfortunately, these studies are referred to as complosomes, and few studies have been reported in which human T cells were used. However, this topic also may be related to xeno immune responses in the future.

## Concerning Non-Gal Antigens

### Gal (GGTA1)

### iGb3 Synthase (GT2)

It was reported that even if GGAT1 is knocked out, GT2 remains and the Gal antigen itself remains abundant on the cell surface. Some teams have actually knocked out both of these antigens (64). However, it appears that the Gal antigen itself is nearly completely eliminated by the KO of GGAT1 (1, 2, 63).

### The H-D Antigen

Since the Hanganutziu-Deicher (H-D) antigen is expressed in Old World monkeys, it has not been extensively addressed in preclinical studies (65–68).

It is also weak as a natural antibody in these KO-mice and is produced after transplantation (69). However, its cytotoxicity has been clearly confirmed. In addition, this antigen is not produced by New World monkeys.

Moreover, recent reports suggest that the KO of Cytidine monophospho-*N*-acetylneuraminic acid hydroxylase (CMAH) increases the antibody titer in monkeys (baboons).

### Sda Antigen

β4GalNT-2 is recognized as a blood group antigen and is expressed by about 90% of humans (70). The issue of whether there is a difference between the expression of Sda in pigs and in humans remains unclear (71).

Blood group antigens are an important issue in transplantation because it is thought that they are expressed on the endothelium of organs. Identifying them and knocking out all of them will be very time consuming.

For example, 90% of pigs have type the A blood group and the remaining 10% have the type O blood group. On the other hand, many of the monkeys used in preclinical experiments contain type B blood.

### The Neoantigen in the GalT-KO Pig

Concerning the neoantigen in the GalT-KO pig, when the KO of Gal was started, there was some hesitation that the knockout procedure would leave its substrate on the porcine carbohydrate chain and it would become a new antigen (2). However, this does not appear to be a problem. the substrate must be expressed by humans, since Gal is knocked out. Moreover, glycosyltransferases are not generally 100% functional and remain as substrates to some extent.

The KO of some genes in pigs, e.g. Gal, may cause changes in the N- and O-glycans of such molecules produced by the pig, resulting in functional changes. However, since the transgenic human molecule does not contain Gal, this does not appear to be a problem.

## Closstalk Between the Complement and Coagulation Systems

Complement and coagulation pathways are closely related (72, 73).

As mentioned above, thrombin acts as a potent C5-converting enzyme that generates C5a, especially in the absence of C3 (74). Similarly, it has been proposed that complement-independent enzymes such as thrombin, neutrophil elastase, and macrophage serine proteases are endowed with C5-converting enzyme activity (75). These pathways should be considered during xenograft rejection.

In addition, coagulation system serine proteases such as Xa, XIa, and plasmin also have the ability to cleave C3 and C5 (76). The von Willebrand factor also interacts with complement components.

It has also been reported that platelets interact with complement and the coagulation system. Activated platelets have been reported to act on factors of the lectin pathway (77) and to bind to plasma proteins to activate factors XIIa and XIa, and kallikrein.

Therefore, under certain circumstances such as AVR/AHXR, serine proteases of the coagulation system may be involved in the cleavage of C3 and C5. Conversely, it is also well known that C5a activates tissue factor (TF) and initiates the coagulation cascade (78, 79). An association with vasculitis has also been noted (4).

## IBMIR and IRI

### IBMIR (The Instant Blood-Mediated Inflammatory Reaction)

This reaction is triggered by platelets, the coagulation system, the complement system of all three pathways, and infiltrates the islets (80) Isolated islets may also serve as indicators of extracellular matrix proteins as new antigens (81). That is, not only tissue factor (TF) expression but also intrinsic pathway activation by collagen and other negatively charged molecules on the islet surface, which are not normally in contact with the blood, due to the procedure that involves islet harvesting (82). At the same time, IRI activates the complement pathway as a matter of course.

Therefore, not only anti-complement drugs but also anticoagulants such as heparin (83) and other molecules have been used to prevent or inhibit this reaction (84). The expression of hCRP is at least required to protect porcine islets from human complement during the reaction (85, 86).

### Ischemia-Reperfusion Injury

In the case of pancreatic islets, this process starts when the islets are transplanted into blood vessels, and in the case of organs, it occurs during reperfusion. As an IRI, this phenomenom is involved in antibody, and all-mediated rejection and the development of thrombic microangiopathy and transplant vasculopathy (87, 88).

Another important aspect of Xeno-rejection is that when pig cells are destroyed, Damage-Associated Molecular Patterns (DAMPs) and Pathogen-Associated Molecular Patterns (PAMPs) are released, which are then phagocytosed by human APCs (Dendric Cells, Mcrophages, etc.). This results in the transmission of information on adaptive immunity (lymphocytes) and is independent of inflammation. This is usually combined with opsonin action of complement.

However, the regulation of these macrophages and others is also a major challenge for the future. A single CD47 (89) molecule is not sufficient, and comprehensive regulation including HLA classIb (90, 91), CD200 (92), TIGIT (93), CD177 (94), etc. needs to be carefully examined.

## Concluding Remarks

In the USA, clinical trials of xenotransplantation have been initiated, using gene-modified pigs. Other teams of the world are also preparing to launch trials.

Complement regulation, along with the regulation of coagulation factors, appears to be the basis for successful xenotransplantation. However, simply expressing two CRPs along with several anticoagulants in pigs without taking their expression levels into consideration may not be sufficient. It may also be necessary to consider both the complement of the pig being released from the graft in addition to liver grafts. Also, the tight control of innate immunity such as macrophages, to control the movement of lymphocytes, will be important as the next step in the era of xenotransplantation is realized.

## Author Contributions

SM organized, researched, discussed, and wrote this review. All other members participated in the research and discussions, especially, MI and HO supervised the project. All authors contributed to the article and approved the submitted version.

## Conflict of Interest

The authors declare that the research was conducted in the absence of any commercial or financial relationships that could be construed as a potential conflict of interest

## Publisher’s Note

All claims expressed in this article are solely those of the authors and do not necessarily represent those of their affiliated organizations, or those of the publisher, the editors and the reviewers. Any product that may be evaluated in this article, or claim that may be made by its manufacturer, is not guaranteed or endorsed by the publisher.
